# Low Energy Availability and Its Impact on Bone Health and Metabolism in Athletes: A Narrative Review

**DOI:** 10.33549/physiolres.935749

**Published:** 2025-12-01

**Authors:** Jan KONVIČKA, Marcela KÁŇOVÁ, Nadezhda BORZENKO, Karin PETŘEKOVÁ, Marek BUŽGA

**Affiliations:** 1 Department of Physiology and Pathophysiology, Faculty of Medicine, University of Ostrava, Ostrava, Czech Republic; 2 Department of Epidemiology and Public Health, University of Ostrava, Ostrava, Czech Republic; 3 Department of Anaesthesiology and Intensive Care Medicine, University Hospital Ostrava, Ostrava and University of Ostrava, Czech Republic

**Keywords:** Energy availability, Relative energy deficiency in sport, Resting metabolic rate, Bone mineral density, Sport

## Abstract

Low energy availability (LEA) is a recognized risk factor that affects the health and performance of athletes. This narrative review summarizes current evidence on the relationship between LEA, resting metabolic rate (RMR), and bone mineral density (BMD). It focuses on the applicability of the RMR ratio as an indicator of metabolic adaptation to energy deficiency and analyzes the associations between energy availability and skeletal health outcomes. This narrative review demonstrates that reduced energy availability is related to a decrease in the RMR ratio and hormonal alterations characteristic of Relative Energy Deficiency in Sport (RED-S). Furthermore, prolonged LEA has been associated with impairments in bone metabolism and lower Z-scores, particularly among endurance and aesthetic athletes. However, the findings also suggest that the impact of LEA on BMD may be modulated by sport-specific loading patterns and additional individual factors. Considerable methodological heterogeneity between studies limits the direct comparability of the results, highlighting the need for standardization in the evaluation of EA, RMR, and BMD. This review emphasizes the importance of comprehensive screening strategies combining nutritional, metabolic, hormonal, and skeletal markers for early identification of the risk of RED-S. Future research should prioritize longitudinal designs to better understand the dynamics of metabolic and skeletal changes in response to fluctuations in energy availability.

## Introduction

Low energy availability (LEA) is a prevalent issue in athletic populations and represents a significant risk factor for impaired physiological functioning [1,2]. LEA occurs when dietary energy intake is not sufficient to cover the energy expended during exercise while maintaining essential physiological processes [1]. Its consequences are encapsulated in the concept of Relative Energy Deficiency in Sport (RED-S), which encompasses a spectrum of health and performance impairments affecting both female and male athletes [2,3].

Among the most concerning outcomes of prolonged LEA are disruptions in bone metabolism and reductions in bone mineral density (BMD) [2]. These effects are especially pronounced in sports with high energy demands, such as endurance and aesthetic disciplines [2]. While hormonal and metabolic adaptations such as decreased resting metabolic rate (RMR), low triiodothyronine (T3), and leptin levels can precede overt clinical signs [4–6], reduced BMD and increased risk of fractures often emerge as long-term consequences of persistent energy deficiency [4,5].

Therefore, early detection of LEA and RED-S is essential. A multifactorial assessment strategy, combining nutritional, hormonal, metabolic, and skeletal markers, has been proposed to improve the identification of athletes at risk [4–6]. Among these, the RMR ratio that compares the measured and predicted RMR has gained attention as a practical indicator of metabolic adaptation to energy deficiency [6,7]. This review focusses on the association between LEA, the RMR ratio, and BMD, with the aim of synthesizing current evidence and identifying key methodological and clinical considerations.

## Resting metabolic rate

Resting metabolic rate (RMR) represents the amount of energy required to maintain basic physiological functions at rest and accounts for the largest portion of daily energy expenditure (approximately 50–70 %). In athletes, RMR measurement can serve as an important tool for identifying low energy availability states (LEA). A decrease in RMR is a typical physiological adaptation to prolonged energy restriction and can indicate the presence of LEA even before clinical signs such as menstrual disturbances or reductions in BMD become apparent [7]. Adaptations to LEA also include endocrine alterations. Hormonal responses to energy deficiency typically involve decreases in triiodothyronine (T3), leptin, and insulin. On the contrary, growth hormone (GH) levels may be elevated, while insulin-like growth factor 1 (IGF-1) levels are generally suppressed, potentially affecting not only metabolism but also bone tissue and recovery capacity [5].

### Metabolic adaptations to changes in energy intake

A short-term energy deficit (lasting less than 14 days) typically causes a mild and reversible decrease in RMR, which tends to normalize once energy availability is restored. This is accompanied by rapid hormonal adaptations, most notably reductions in leptin and T3 levels, indicating an acute downregulation of metabolic activity aimed at preserving energy stores [6].

On the contrary, a chronic energy deficit (lasting more than 14 days) leads to more pronounced and sustained reductions in RMR. This is accompanied by significant and persistent decreases in T3, leptin, and IGF-1 levels. These hormonal changes may have more serious health consequences, such as impairments in bone mineral density, muscle mass, and immune function [5,8,9].

A short-term energy surplus increases RMR due to elevated insulin and leptin levels, along with a modest increase in T3. These hormonal changes improve metabolic rate and energy expenditure, which can be strategically used in athletic settings to support recovery and performance [6]. A prolonged energy surplus leads to a consistently elevated RMR. When accompanied by increased physical activity and training, it can promote gains in fat-free mass and further increase RMR [6]. This effect is generally observed when energy availability exceeds 45 kcal/kg FFM/day [2]. However, a chronic energy surplus without adequate physical load can result in undesirable increases in fat mass.

### Predictive equations for resting metabolic rate

Reductions in RMR due to chronic LEA are often more pronounced than would be expected based solely on changes in body weight or body composition. For this reason, predictive equations are commonly used to estimate expected RMR and assess deviations in measured values. Widely used formulas, such as the Harris-Benedict or Mifflin-St Jeor equations, may underestimate actual RMR in athletic populations, as they do not account for specific characteristic of body composition-particularly the higher proportion of fat-free mass (FFM), which is more metabolically active [7,10]. Therefore, it is recommended that predictive equations for athletes directly incorporate FFM. Selecting an appropriate prediction model that reflects both FFM and sport-specific characteristics is essential to estimate expected RMR [11].

An important concept for evaluating the metabolic consequences of LEA is the RMR ratio, defined as the ratio between measured and predicted RMR. An RMR ratio below 0.90 indicates that the measured RMR is significantly lower than expected and may serve as a useful marker to identify athletes at risk for chronic energy deficiency and its associated health consequences. The RMR ratio is also associated with hormonal changes, particularly in T3 and leptin, which are closely related to energy availability and metabolic state [6,7].

## Bone health

Bone tissue is a dynamic organ that continuously undergoes remodelling through the coordinated activity of two primary cell types – osteoblasts (bone formation) and osteoclasts (bone resorption). The balance between these processes is critical for maintaining bone health. Reduced energy availability disrupts this balance, primarily through changes in endocrine regulation. LEA leads to decreased levels of key hormones involved in bone metabolism, especially estrogens, IGF-1, and leptin while increasing cortisol levels [4,5,12]. Estrogen plays a pivotal role in bone preservation. Its deficiency, resulting from LEA-induced disruption of the hypothalamic-pituitary-gonadal axis, increases osteoclast activity and reduces osteoblast activity, thereby accelerating bone loss [13].

IGF-1 is a key stimulator of osteoblast function. Animal studies have shown that dietary restriction significantly reduces serum IGF-1 levels and leads to cortical bone loss [12]. This reduction in IGF-1 directly affects the ability of osteoblasts to form new bone, thus further disrupting remodelling. Leptin, secreted by adipose tissue, is involved in the central regulation of energy balance. Low levels of leptin under energy-deficient conditions not only suppress resting energy expenditure (REE), but also affect bone metabolism by inhibiting osteoblast activity [4]. Conversely, elevated levels of cortisol under chronic physical or energy stress stimulate bone resorption by improving osteoclast activity and suppressing bone matrix formation [4,14].

LEA affects bone metabolism not only through alterations in core hormones such as estrogen, IGF-1, leptin, and cortisol, but also through modulation of vitamin D status and its interaction with other bone-related factors. Vitamin D plays a crucial role in calcium homeostasis and bone metabolism. Its deficiency is associated with an increased risk of stress fractures and osteoporosis not only in older adults, but also in young athletes [5,12]. The therapeutic administration of active vitamin D analogues for the purpose of protecting bone health or restoring IGF-1 levels as a result of LEA remains unconfirmed. Some studies suggest that calcium and vitamin D supplementation reduces the incidence of stress fractures in young athletes [4,5]. A review from 2021 recommends maintaining serum vitamin D levels above 32 ng/ml while not exceeding the normal range, together with ensuring adequate calcium intake, to reduce the skeletal impact of LEA in hypogonadal male athletes with low BMD [5].

In addition to vitamin D and the aforementioned hormones, parathyroid hormone (PTH) also plays an important role. Its serum concentration is inversely affected by vitamin D status. A deficiency in vitamin D leads to increased PTH, which in turn can enhance bone resorption and worsen bone microarchitecture. Thus, sufficient levels of vitamin D help prevent bone loss [4,5].

### Evaluation of bone health

Bone mineral density (BMD) is most commonly assessed using imaging techniques. The standard method is dual-energy X-ray absorptiometry (DXA), which allows not only the evaluation of total BMD but also the separate analysis of the trabecular and cortical bone compartments [15]. Recently, 3D-DXA technology has emerged, offering a more detailed evaluation of the bone microarchitecture. This is particularly important, as BMD alone may not fully reflect actual bone strength [15].

BMD is typically measured at standardized anatomical sites [16], such as:

- TBMD (Total Bone Mineral Density): whole-body bone density,
- LBMD (Leg Bone Mineral Density): density in the lower limbs, which are subject to the highest mechanical load,
- LSBMD (Lumbar Spine Bone Mineral Density): lumbar spine density, often sensitive to changes in energy availability and hormonal status.

BMD is evaluated using T-scores (comparison with a young healthy population) and Z-scores (comparison with an age-matched population). In athletes, especially those in high-impact disciplines, standard BMD values are often elevated compared to the general population. Therefore, the interpretation of the Z-score in athletes must be individualized and account for sport-specific characteristics. A Z-score <−1.0 is already considered indicative of compromised bone Health [17].

### Clinical consequences

The clinical consequences of long-term low energy availability and subsequent disturbances in bone metabolism include reduced BMD and an increased risk of stress fractures. Low BMD is directly associated with a higher incidence of stress-related fractures, which are commonly observed in endurance runners and ballet dancers, for example. In some cases, BMD values may remain within normal limits despite the persistence of LEA-related symptoms [15,16]. Prolonged LEA not only leads to acute health problems such as stress fractures, but also contributes to the long-term deterioration of the bone microarchitecture, increasing the risk of osteopenia or osteoporosis later in life [5,12].

Athletes with LEA often exhibit symptoms of RED-S, which include not only compromised bone density, but also other health and performance impairments resulting from insufficient energy intake [5,16].

In clinical practice, emphasis should be placed on early diagnosis and prevention. This includes a regular assessment of energy availability, hormonal profiles, and bone density using DXA or 3D-DXA [5,15].

## Methods

**Fig. 1:**
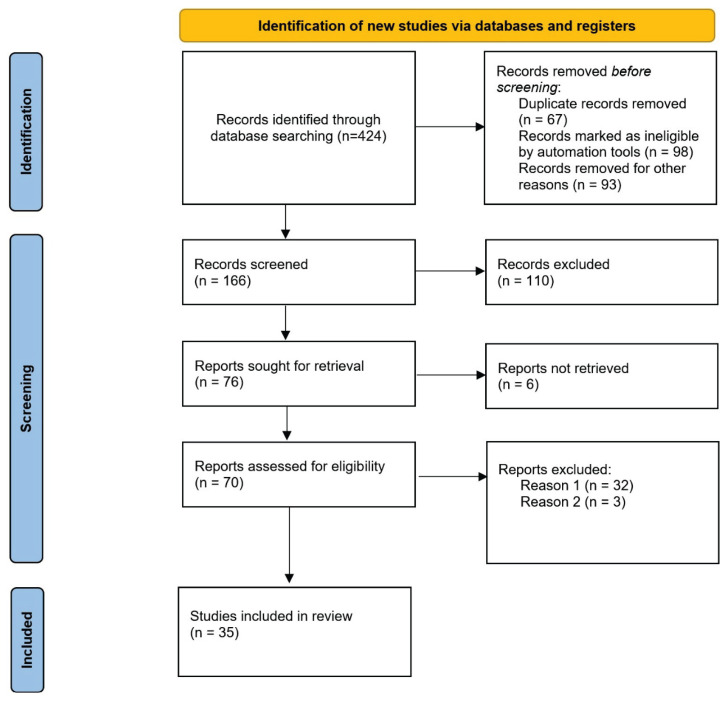
PRISMA flow diagram of study selection.

This narrative review aims to explore the current understanding of how low energy availability affects bone health and metabolic function in athletes, with a specific focus on bone mineral density and suppression of resting metabolic rate. The objective of this review is to synthesize findings from the recent literature that investigate the physiological consequences of LEA, particularly reductions in the RMR ratio and alterations in bone density, in different athletic populations. Relevant studies were identified through a structured search of the PubMed and Web of Science databases, according to accepted guidelines for the PRISMA guidelines (Fig. 1).

Search terms included: (“low energy availability” OR “RED-S”) AND (athletes OR “female athletes“ OR “male athletes” OR “adolescent athletes” OR sport) AND (“bone mineral density” OR osteopenia OR osteoporosis OR “stress fracture” OR “resting metabolic rate” OR RMR OR BMR OR “metabolic adaptation” OR “basal metabolic rate” OR DXA)

The inclusion criteria were as follows:

1. The study includes measurement of BMD or RMR ratio.
2. The study calculates energy availability or using screening questionnaires related to LEA.
3. The study participants are athletes or members of the active population.

Additional articles were included by screening reference lists of relevant articles. Duplicates were removed, and the abstracts were reviewed for relevance.

## Low energy availability and athlete metabolism

The severity of energy deficiency is often evaluated using the RMR ratio. A value below 0.90 indicates significant metabolic adaptation [6,7]. In our review, we compared the results of studies that evaluated the RMR ratio in relation to LEA using both objective calculations of EA and questionnaire-based methods (e.g., LEAF-Q or EDE-QS) summarized in Table 1.

Melin *et al*. [18], in an observational study of endurance athletes, identified a significantly reduced RMR ratio (0.87±0.06) in the low EA group (19.1 kcal/kg FFM/day), with the reduction being statistically significant (p=0.047). Even suboptimal EA (38.5 kcal/kg FFM/day) was associated with a downward trend in the RMR ratio to 0.87±0.07 (p=0.08). On the contrary, optimal EA (51.7 kcal/kg FFM/day) was associated with a normal RMR ratio (0.93±0.07), suggesting that metabolic adaptations can occur even with mildly reduced EA and worsen as the energy deficit deepens [18].

Similarly, in the RCT by Oxfeldt *et al*. [19], a significant decrease in resting metabolic rate and T3 concentration was observed when EA was reduced to 25 kcal/kg FFM/day, confirming the sensitivity of metabolic regulation to energy deficits. In contrast, an optimal EA of 50 kcal/kg FFM/day did not result in significant changes, supporting the findings of Melin *et al*. [18].

Comparable results were also found among ballet dancers. Staal *et al*. [11] reported a significantly reduced RMR ratio (<0.90) in dancers with low EA (<30 kcal/kg FFM/day), while athletes with greater energy availability maintained RMR within normal limits [11].

In a cross-sectional study, Torstveit *et al*. [20] found a markedly reduced mean RMR ratio (0.86±0.07) in male endurance athletes, with 72 % of the athletes exhibiting a ratio ≤0.90. These findings highlight the high prevalence of metabolic adaptations induced by prolonged LEA in all sports disciplines and also in male athletes [20].

Questionnaire-based methods of LEA assessment methods also demonstrate considerable sensitivity in detecting reduced RMR. In the study by Moris *et al*. [21], conducted among collegiate athletes (basketball, golf, soccer, wrestling, and cross-country), a high prevalence of a reduced RMR ratio (≤0.90) was reported, ranging from 40 % to 75 % depending on the sport. These findings suggest that tools such as the LEAF-Q can serve as effective screening methods for the early detection of athletes at risk for LEA [21].

Uriegas *et al*. [22], using the EDE-QS questionnaire, also identified a high prevalence of reduced RMR (60 %≤0.90) among female athletes of collegiate level, indicating a strong link between energy availability, disordered eating, and metabolic adaptations [22]. Similar conclusions were drawn by Vardardottir *et al*. [23], who found a significant prevalence of LEA combined with low carbohydrate intake and elevated disordered eating scores, further increasing the risk of a marked decline in RMR [23].

## Low energy availability and bone mineral density in athletes

The negative effects of low energy availability (LEA) on bone mineral density (BMD) are well documented in the scientific literature. Since bone mass development and maintenance are also influenced by the type of physical activity, it is important to consider sport-specific loading when assessing the risk of poor bone health. A summary of the collected data is displayed in Table 2 and Table 3.

In a cross-sectional study of college athletes from various disciplines, Moris *et al*. [21] reported a high prevalence of LEA as determined by the LEAF-Q questionnaire and low Z-scores, particularly among golfers and runners. These athletes also exhibited an increased risk of bone injuries. On the contrary, wrestlers, although also having a high prevalence of LEA, showed relatively high BMD values (Z-scores), suggesting that the type of mechanical loading in sport may play a protective role in preserving bone mass even in the presence of low energy availability [21].

Similarly mixed findings were reported by Rogers *et al*. [24], who analyzed 112 female athletes in different team and endurance sports (e.g., basketball, triathlon, water polo, rowing). Based on LEAF-Q, 55 % of athletes met the criteria for LEA (score ≥8). Regarding the skeletal parameters, 3 % had a Z-score for the lumbar spine (LS) below -1.0, and 1 % had a Z-score below −2.0. However, the data were not stratified by sport type [24]. In a subsequent study, Rogers *et al*. [25] again used the LEAF-Q and found that 55 % of 75 female athletes in various disciplines were at risk of LEA. Although the average Z-scores were positive, there was high variability: 0.5±1.2 for the LS and 0.8±1.2 for the femoral neck (FN). The control group (n=31), with LEAF-Q scores <8, exhibited higher Z-scores [25].

The ED-SQ questionnaire has also been proven to be useful in identifying the skeletal risk associated with LEA. Holtzman *et al*. [26], in their study of young female athletes, found an increased prevalence of low BMD (Z-score <-1) among those with RED-S risk factors. These findings support the use of screening tools and clinical evaluations to identify bone health risks related to LEA [26].

The detrimental impact of LEA on bone health is also documented among adolescents. In a study by Barrack *et al*. [27], adolescent female athletes with clinical indicators of LEA (amenorrhoea, underweight) showed significantly lower Z-scores in the LS (−1.30±1.38) and total body (TB) (−0.30±0.98) compared to controls. Interestingly, most athletes did not report intentional dietary restriction, suggesting a major role of unintentional energy deficiency in the development of low BMD [27].

LEA may also be indicated by discrepancies between actual and ideal body weight. Nose-Ogura *et al*. [28] defined LEA in adolescent female athletes as having an ideal body weight (IBW) <85 %, and in adult women as having a BMI ≤17.5. The prevalence of LEA was 14 %, and 22.7 % of athletes had a LS Z-score <−1. However, only 0.7 % of the athletes had both LEA and a Z-score <−1, suggesting that defining LEA solely by IBW or BMI may be insufficient [28].

## Endurance sports

### Calculated Energy Availability (EA)

Heikura *et al*. [29] demonstrated significantly lower BMD values (1.164±0.041 g/cm^2^; p<0.05) in female endurance athletes with amenorrhoea (EA: 32±12 kcal/kg FFM/day), compared to their eumenorrheic counterparts (EA: 35±9 kcal/kg FFM/day; BMD: 1.222±0.076 g/cm^2^). Consequently, the Z-score in the amenorrheic group was significantly lower (−0.3±0.9; p<0.05), suggesting that even a moderate reduction in energy availability can have clinically relevant effects on bone health. A higher incidence of bone injuries was also observed in this subgroup. However, among male athletes, no significant differences in BMD were observed between those with reduced testosterone levels (TES: 15.1±3.0 nmol/l) and those with normal levels (TES: 25.0±7.1 nmol/l), despite similar EA values, suggesting a potential protective role of testosterone in bone metabolism under LEA conditions [29].

Further evidence from Gama *et al*. [30] indicated that female long-distance triathletes with EA<45 kcal/kg FFM/day exhibited lower cortical bone density, area, and thickness compared to athletes with sufficient energy availability [30].

Kinoshita *et al*. [31] stratified adolescent female middle- and long-distance runners into three groups based on EA levels. Interestingly, the group with the lowest EA (15.5±6.2 kcal/kg FFM/day) showed a slightly higher Z-score (0.4±0.3) compared to both the intermediate (Z=0.1±0.5) and high EA groups (Z=−0.1±0.5). This counterintuitive finding may reflect sample size limitations (n=6), short-term duration of energy restriction, or interindividual biological variation. However, the prevalence of amenorrhoea reached 66.7 % in the low EA group, which aligns with other findings related to RED-S [31].

Moore *et al*. [32] reported no cases of low BMD among male endurance athletes despite the presence of LEA. However, carbohydrate intake was markedly reduced (<5 g/kg in 89 % of athletes), and testosterone levels were affected. Although these changes did not directly impact BMD in this cohort, they may indicate broader metabolic and reproductive risks, especially under prolonged energy restriction [32].

On the other hand, Haines *et al*. [33] found that male distance runners with EA<38 kcal/kg FFM/day had significantly lower Z-scores (−1.76±0.75) compared to athletes with EA>38 kcal/kg FFM/day (−0.75±0.81), supporting the link between inadequate energy availability and altered bone status [33].

In a recreational endurance population, Lane *et al*. [34] identified LEA in 61.7 % of athletes. Despite overall positive Z-scores (0.73±0.95 overall; 1.46±1.36 LS; 1.59±1.17 FN), a significant inverse correlation was found between EA and BMD (r=−0.360, p=0.004) [34]. These findings suggest that subclinical effects of LEA on skeletal health may occur even in non-elite populations with seemingly normal bone parameters.

### Questionnaire-based LEA assessment in endurance athletes

Several studies have employed validated questionnaires as an alternative or complement to direct EA measurement, particularly in endurance disciplines where detailed tracking of dietary and expenditure may be impractical. These approaches improve the detection of RED-S and are especially valuable when objective EA measurement is not feasible.

Skorseth *et al*. [35] reported a prevalence of disordered eating of 76.3 % among adolescent long-distance runners using the EDE-Q, with 42.1 % of participants presenting a Z-score <−1. These results indicate a strong relationship between eating behavior, low energy availability, and compromised bone health during critical developmental years [35].

Using the SEAQ-I (Sport-specific Energy Availability Questionnaire and Interview), Keay *et al*. [36] identified LEA in 28 % of competitive male cyclists. In particular, 44 % of these athletes also exhibited LS Z-scores <−1.0 (p<0.001), with EA emerged as the primary determinant of BMD in this region [36].

Kyte *et al*. [37] compared elite female distance runners with a control group using LEAF-Q screening. The risk of LEA was present in 47 % of runners versus 13 % of controls. While runners had significantly higher TB Z-scores (1.7 [1.2–2.3] vs. 0.9 [0.8–1.0], p<0.001), their leg bone Z-scores were lower, although not significantly. This suggests that reduced BMD may manifest itself in specific skeletal regions depending on sport-specific loading patterns [37].

Gimunová *et al*. [38] examined a cohort of recreational female endurance athletes, reporting a prevalence of LEA based on LEAF-Q of 20–30 %. The TB Z-scores ranged from −0.5 to −1.1, with more negative values observed among those with higher LEAF-Q scores. These findings indicate that even in nonelite populations, questionnaire tools can effectively identify people at increased risk of bone demineralization [38].

Mathisen *et al*. [14] applied the RED-S CAT scoring in women engaged in recreational endurance-based fitness activities and found that 47 % were at risk for RED-S. These athletes showed lower Z-scores (e.g., −0.8 vs. +0.2 in non-risk individuals) and commonly presented with other RED-S indicators such as amenorrhoea, low energy intake, and reduced leptin concentrations [14].

## Aesthetic Sports

Aesthetic disciplines are associated with an exceptionally high prevalence of low energy availability (LEA), often involving severe deficits compounded by strict demands on body composition. In professional ballet dancers with extremely LEA (0.6 to 5.8 kcal/kg FFM), Doyle-Lucas *et al*. [39] reported Z-scores ranging from 0.73 to −0.25. Amenorrhoea was present in 50 % of the participants, while the rest experienced irregular or oligomenorrheic cycles. Psychological screening (TFEQ, EAT-26) indicated restrictive and problematic eating behaviors in dancers with the lowest energy availability. Despite relatively high Z-scores in some individuals, energy availability was markedly lower in ballet dancers (3.75±2.2 kcal/kg FFM) compared to the control group (41.1±4.6 kcal/kg FFM), whose average Z-score was 1.20±0.3. Among dancers, regardless of the status of EA, the mean Z-score reached 0.99±0.2. However, the presence of suppressed RMR, amenorrhoea, and oligomenorrhea highlights clinical indicators of RED-S, even in cases where BMD remains within normal reference ranges [39].

Civil *et al*. [40] provides more evidence, reporting an average EA of 39.5±10.8 kcal/kg FFM in a sample of ballet dancers, with 22 % falling below the critical threshold of 30 kcal/kg FFM. According to the LEAF-Q questionnaire (cut-off ≥8), 65 % of the participants were identified as being at risk of LEA. Despite the average EA exceeding the clinical threshold, the occurrence of menstrual disturbances (25 % secondary amenorrhoea, 15 % oligomenorrhea) and a mean Z-score of the LS of 1.145±0.930 indicate that even marginal energy deficiency may compromise hormonal and skeletal homeostasis in this population. These findings point to a latent risk of skeletal complications despite apparently normal BMD [40].

Staal *et al*. [11] also reported that although the prevalence of low bone mineral density (Z-score ≤-1) among ballet dancers was relatively low (5 %), higher LEA symptom scores, particularly those reflecting reduced energy intake were associated with lower BMD values [11].

Additional evidence of a high prevalence of LEA comes from Smith *et al*. [41], who investigated competitive cheerleaders and found extremely low mean levels of EA (12.48±8.01 kcal/kg FFM). More than half of the athletes (52.6 %) reported menstrual irregularities and met self-reported criteria for the risk of disordered eating, while 14.2 % had hormonally confirmed amenorrhoea. In particular, the average Z-score was 1.7±0.74, well above normative values. This mismatch suggests that Z-scores may lack sensitivity to detect short- or medium-term consequences of LEA or that, in young athletes with genetic advantage, transient LEA may not immediately compromise bone density [41].

Contrasting findings were observed by Besor *et al*. [42], who, in a small sample of acrobatic gymnasts, reported an elevated prevalence of low Z-scores in conjunction with the risk of LEA as assessed by the DEAQ [42].

Ikegami *et al*. [43] provide further information, who evaluated track and field athletes and gymnasts and reported a mean EA of 31.8±15.0 kcal/kg FFM slightly above the LEA threshold, but substantially lower in some cases due to high variability. In this study, LEA was estimated using deviations from ideal body weight, a method that can reduce the accuracy of the actual EA assessment. The reported Z-scores (total body less head: 0.53±0.75; lumbal spine: 0.40±0.90) indicate suboptimal bone density despite the osteogenic nature of these sports. A history of amenorrhoea reported in 14.3 % of participants once again highlights the potential long-term adverse effects of reduced energy availability, even when bone health markers remain within a seemingly acceptable range [43].

## Strength-focused sports

Among strength-based disciplines, the most relevant findings come from Mursu *et al*. [44], who examined athletes engaged in physical sports and gym-based resistance training (including bodybuilding, fitness, and recreational strength training). The prevalence of LEA reported ranged from 0 to 26 %, depending on the subgroup. The average EA values ranged from 35.3 to 41.3 kcal/kg FFM consistently above the LEA threshold, with all subgroups showing Z-scores within the normal range. No clinically significant osteopenia was observed in any subgroup, which may reflect the protective effect of resistance training through increased mechanical loading on the skeleton, a factor known to enhance bone strength and potentially offset other physiological stressors. The authors emphasized that although metabolic and hormonal markers were not always optimal, the characteristic high mechanical load of strength sports can provide skeletal protection, which could explain the preservation of normal Z-scores even in the presence of suboptimal reproductive health indicators [44].

Vardardottir *et al*. [23] provide additional information, including female athletes from both strength and weight-class sports. In the subgroup with the lowest EA (23.3±5.5 kcal/kg FFM), Z-scores remained relatively high (1.4±0.9), however this was accompanied by clinical signs such as amenorrhoea, oligomenorrhea, and elevated EDE-QS scores indicative of the risk of disordered eating. Other subgroups reported EA levels above 30 kcal/kg FFM and Z-scores ranging from 1.1 to 1.7, with no reported menstrual dysfunction. Interestingly, even in the lowest EA group, Z-scores did not fall below normal limits, supporting the hypothesis that the relationship between EA and bone density in strength sports may be attenuated or modulated by chronic mechanical loading. However, the presence of amenorrhoea and eating disorders in this subgroup underscores the persistence of health risks beyond bone Health [23].

This pattern is echoed in the findings of Edama *et al*. [45], who studied athletes specializing in throws, jumps, and other power events. No cases of low energy availability (LEA) were reported, and no athlete had a Z-score below −1.0. Similarly, amenorrhoea was absent in this cohort [45].

## Team sports

In the context of team sports, Moss *et al*. [46] provided important data from a cohort of female football players. The average energy availability was 35±10 kcal/kg FFM. Based on the LEAF-Q questionnaire (cut-off ≥8), 23 % of athletes were classified as at risk for LEA. Suboptimal EA was observed in 62 % of the players, while only 15 % met the criteria for optimal availability. Despite this, the average Z-score was notably high: 2.4±0.9, with amenorrhoea reported in just 8 % of the participants. This discrepancy between a high prevalence of LEA symptoms and elevated Z-scores may reflect compensatory factors such as skeletal loading from sport-specific movements, adaptive training effects, or the short duration of energy deficiency. EA was positively correlated with BMI and body fat percentage [46].

These findings are supported by Edama *et al*. [45], who analyzed athletes from three team sports: football, basketball, and volleyball. In all groups, Z-scores were within normal limits, and LEA prevalence was exceptionally low, with at most one case identified per sport [45].

Similarly, Łuszczki *et al*. [47] evaluated 22 female football players and reported that 64.7 % screened positive for the risk of LEA using the LEAF-Q. Although only 6.06 % of the participants reported current amenorrhoea, up to 24.24 % had experienced it in the past. However, the average Z-scores remained favorable (1.15±0.85), aligning with other research indicating that football players generally maintain a strong skeletal profile despite some functional disturbances [47].

A particularly insightful series of studies comes from Zabriskie *et al*. [48], who monitored collegiate lacrosse players throughout different phases of the season. In the early season phase, the EA was 30.4±11.0 kcal/kg of FFM, with a Z-score of 1.30±0.76. As the season progressed, the EA decreased – to 26.2±10.5 in phase II, and further to 22.9±8.5 kcal/kg FFM in phase III. During the postseason, EA increased again slightly to 28.9±9.2 kcal/kg FFM, while Z-scores reached their maximum at 1.46±0.75 [48].

In contrast, Kalpana *et al*. [49] examined male athletes competing in the traditional Kho-Kho sport and found a markedly low EA (14.62±5.21 kcal/kg FFM/day). This was associated with significantly reduced Z-scores (−0.60±0.71; p<0.05), indicating the detrimental impact of extremely low energy availability even in male athletes. These findings are particularly relevant given the often overlooked risk of LEA in men, where its clinical significance tends to be underemphasized [49].

## Discussion

Based on the reviewed studies, it is evident that both objectively measured energy availability (EA) and questionnaire-based low energy availability (LEA) assessments are strongly associated with a suppressed resting metabolic rate (RMR) ratio, supporting their clinical and practical relevance for the early identification of energy deficiency and associated health risks in athletes. However, certain limitations must be acknowledged. Methodological heterogeneity, including differences in EA calculation versus questionnaire-based screening, varied predictive equations for RMR, and inconsistent sample sizes limits the generalizability of findings. Future research should clearly define methodological procedures for RMR assessment, apply standardized criteria for LEA diagnosis, and include larger and more homogeneous athletic samples to strengthen the validity of conclusions. The RMR ratio represents a key indicator of metabolic adaptations to low energy availability and should be routinely incorporated into comprehensive nutritional assessments of athletes. Its integration may significantly improve the early detection and prevention of Relative Energy Deficiency in Sport (RED-S) and other health complications associated with chronic LEA. An important consideration is the appropriate selection of predictive equations tailored to specific sports, as commonly used formulas often produce substantial inaccuracies in RMR estimation [11].

The overview of existing literature that examines the relationship between EA and bone mineral density (as reflected in Z-scores) further underscores the complexity and methodological variability of identifying and interpreting LEA in athletic populations [21,27,29,36]. Although most studies confirm a general association between low EA and impaired skeletal markers, the broader picture is multifactorial and highly dependent on the type of sport, the EA assessment method, the diagnostic tools used, and the presence of clinical symptoms associated with RED-S. Among sport-specific categories, endurance and aesthetic sports consistently emerge as the most at risk, with many studies reporting direct cases of osteopenia (Z-score <−1.0) or statistically significant negative associations between EA and Z-score [11,29,33–36,39,42].

One of the major challenges in current RED-S and EA research is the variability between sports disciplines, reflecting not only divergent nutritional demands but also distinct physiological responses to energy deficiency. For example, strength athletes may maintain normal Z-scores despite a moderate energy imbalance due to the osteoprotective effects of high mechanical loading, while aesthetic and endurance athletes may experience pronounced reproductive and skeletal dysregulation even with relatively mild deficits. Similarly, team sports findings suggest that a high prevalence of questionnaire-based LEA does not always coincide with impaired bone health possibly due to more adequate fueling, a varied training load, or shorter exposure to energy deficiency. These intersport differences emphasize the need for methodological contextualization: the validation and standardization of RED-S assessment tools must account for sport- and population-specific factors. Applying a universal approach (e.g., a single LEAF-Q cut-off point across all disciplines) without considering such nuances may result in false positives or false negatives [25,44,46,47]. This applies equally to the interpretation of Z-scores, which should be evaluated in relation to loading patterns, hormonal profile, and potential comorbidities.

Although Z-scores are objective and clinically validated, they may not reliably detect RED-S in all athletes, particularly in the early stages or among younger individuals. Similarly, a positive LEA screening using questionnaires such as LEAF-Q, EA-FQ, or RED-S CAT does not necessarily imply skeletal compromise, as evidenced by findings in football players or physique athletes [44,46]. This reinforces the conclusion that no single tool is sufficient in isolation; rather, a combination of nutritional, hormonal, metabolic, and skeletal markers is essential.

From a methodological perspective, there is notable inconsistency between studies in how EA is determined. Some use direct calculations based on energy intake and expenditure, while others rely exclusively on self-reported screening tools (LEAF-Q, RED-S CAT, EDE-QS). These methods are not always comparable and may be influenced by subjectivity, imprecise estimates of energy expenditure, and seasonal variation. Furthermore, many studies lack standardized protocols for the assessment of BMD or do not report the specific anatomical site for Z-score measurements, complicating comparisons between findings.

The available evidence also points to several practical recommendations for clinical care and prevention. In high-risk groups, particularly female athletes in aesthetic and endurance sports, routine monitoring should include not only Z-scores but also menstrual function and metabolic markers such as RMR, leptin, T3, and IGF-1. In athletes without amenorrhoea but with positive screening for LEA, proactive education, surveillance, and intervention should be considered before bone density begins to decline. For male athletes, who often lack overt symptoms, monitoring testosterone levels, mood, recovery, frequency of injury, and body composition changes may offer critical information. From a research perspective, there is an urgent need to standardize EA assessment protocols.

Many studies diverge in how they define measured EA, some calculations fail to account for metabolic adaptation, while others rely on underreported dietary intake [6,11,23]. Furthermore, there is no unified validation approach for questionnaires across sport types; for example, the LEAF-Q was developed for endurance athletes and may not be fully applicable to team or strength-based sports [25,44,46,47].

Future research should prioritize long-term longitudinal studies examining fluctuations in EA and their relationship to BMD and Z-scores over time. Furthermore, the sensitivity and specificity of screening tools should be evaluated in relation to objective outcomes, not only bone metrics but also biochemical and hormonal indicators. Special attention should be directed toward populations with atypical loading profiles, such as paraathletes or adolescent athletes, where standard RED-S models may not apply.

## Conclusions

Low energy availability (LEA) significantly affects metabolic function and bone health in athletes. Suppressed resting metabolic rate (RMR), particularly with an RMR ratio below 0.90, serves as an early marker of energy deficiency and can precede the clinical signs of RED-S. Prolonged LEA is associated with impaired bone metabolism through hormonal changes and increased bone resorption, leading to reduced Z-scores, especially in endurance and aesthetic athletes, where menstrual dysfunction and disordered eating are common.

Although strength and team sport athletes may show preserved bone density despite LEA, likely due to mechanical loading and shorter deficit duration, such protection is not universal. Methodological inconsistencies in EA, RMR, and BMD assessment limit comparability between studies and underscore the need for standardized sport-specific tools. Importantly, predictive equations for RMR should be better specified and adapted to the athlete’s sport and physiological characteristics to improve accuracy. Psychological factors such as restrictive eating and compulsive exercise also contribute to the risk and should be routinely evaluated.

Effective identification and prevention of RED-S require a multi-marker approach combining metabolic, hormonal, skeletal, and behavioral indicators. Future research should focus on longitudinal monitoring of EA, RMR, and BMD, and on validating screening tools across athletic populations, with attention to under-represented groups and psychological drivers of energy deficiency.

## Figures and Tables

**Table 1:** Dependence LEA and RMR ratio.

*Study*	Population (n, age, gender, sport)	Category of LEA (kcal/kg/FFM)	RMR ratio	Notes
*Oxfeldt et al. [19]*	n=15, age 24±3, females, trained	50 (optimal)	unchanged (n.s.)	protein 2.2 g/kg FFM
	n=15, age 26±3, females, trained	25 (low)	decreased by 65 kcal (p<0.0001)	decrease of REE and T3

*Melin et al. [18]*	n=15, age 26.3±5.7, females, triathlon	51.7 (optimal)	0.93±0.07 (n.s.)	
	n=17, age 26.3±5.7, females, triathlon	38.5 (suboptimal)	0.87±0.07 (p=0.08)	trend of decrease RMR
	n=8, age 26.3±5.7, females, triathlon	19.1 (low)	0.87±0.06 (p=0.047)	significant reduction RMR, r=0.31

*Moris et al. [21]*	n=4, age 20.8±0.5, males, basketball	15 % EA<20	75 %≤0.90	
	n=5, age 20.6±0.9, males, golf		40 %≤0.90	lower Z-score
	n=7, age 20.7±0.3, males, football		57 %≤0.90	
	n=10, age 19.6±0.5, males, wrestling		40 %≤0.90	highest Z-score in between groups
	n=5, age 20.6±0.6, males, cross-country		nonspecified	the lowest Z-score

*Lane et al. [34]*	n=60, 43.4±11.6, males, recreational endurance athletes	28.7±13.4	0.99±0.08	

*Uriegas et al. [22]*	n=27, 19±1, females, variety of sports	15.9±10.1 (EDE-QS)	60 %≤0.90	

*Vardardottir et al. [23]*	n=8, 20.4, females, variety of sports	23.3±5.5 (EDE-QS)	nonspecified	RED-S score above cut-off

*Torstveit et al. [20]*	n=53, 35.3±8.3, males, triathlon	37.7±10.9	0.86±0.07 (72 %≤0.90)	

**Table 2:** Calculated LEA and BMD.

*Study*	Population (n, age, gender, sport)	LEA calculated (FFM/kg/FFM)	Z-score
*Heikura et al. [29]*	n=13, 23.8±4.4; females; endurance running and walking	32±12	−0.3±0.9 (p<0.05)
	n=22, 26.7±2.9; females; endurance running and walking	35±9	0.3±0.8
	n=10, 27.4±3.4; males; endurance running and walking	31±12	0.3±0.7
	n=14, 26.9±4.5; males; endurance running and walking	35 ± 5	0.3±0.9

*Kalpana et al. [49]*	n=24, 23.63±3.77; males; Kho-Kho	14.62±5.21	−0.60±0.71 (p<0.05)
	n=28, 22.64±3.64; males; Kho-Kho	51.67±13.69	0.048±0.99 (p<0.05)

*Haines et al. [33]*	n=9, 24.8±4.0; males; endurance running	<38	−1.76±0.75
	n=10, 24.8±4.0; males; endurance running	>38	−0.75±0.81
	n=19, 24.8±4.0; males; control		−0.8±0.8

*Moris et al. [21]*	n=5, 20.6±0.9; males; golf	15 % (EA<20 )	−0.29±0.43
	n=7, 20.7±0.3; males; football		1.54±0.33
	n=10, 19.6±0.5; males; wrestling		2.14±0.27

*Lane et al. [34]*	n=60, 43.4±11.6; males; triathlon	28.7±13.4	0.73±0.95 (TB); 1.46±1.36 (LS); 1.59±1.33 (FN)

*Gama et al. [30]*	n=12, 37.2 (24.7–52.4); females; triathlon (long trail)	<45	FN: 0.2; total hip: 0.3; LS: −0.1
	n=11, 37.3 (28.6–45.7); females; triathlon (long trail)	>45	FN: −0.4; total hip: −0.1; LS: −0.1

*Mathisen et al. [14]*	n=26, 24.4±3.5; females; recreational sport	38.6±11.6	0.42±0.61
	n=9, 25.2±4.2; females; fitness	21.0±2.3	−0.18±0.76
	n=8, 25.2±4.2; females; fitness	29.6±2.1	0.14±0.73
	n=8, 25.2±4.2; females; fitness	38.8±4.8	0.38±0.63

*Zabriskie et al. [48]*	n=20, 20.4±1.8; females; lacrosse (1. phase – September)	30.4±11.0	1.30±0.76
	n=20, 20.4±1.8; females; lacrosse (5. phase – May)	28.9±9.2	1.46±0.75

*Kinoshita et al. [31]*	n=6, 16.8±0.9; females; running (800–5000 m)	15.5±6.2	0.4±0.3
	n=12, 16.8±0.9; females; running (800–5000 m)	42.7±11.1	−0.1±0.5

*Smith et al. [41]*	n=19, 20.2±1.24; females; cheerleading	12.48±8.01	1.7±0.74

*Mursu et al. [44]*	n=50, 27.7±4.1; females; Physique athletes	41.3	within normal range
	n=19, 26.4±4.2; females; Gym enthusiasts	39.4	within normal range
	n=11, 28.0±5.6; males; Physique athletes	37.2	within normal range; 1 PA<−1.0
	n=9, 32.0±4.7; males; Gym enthusiasts	35.3	within normal range

*Ikegami et al. [43]*	n=21, 14.3±0.7; females; athletics and gymnastics	31.8±15.0	TBLH: 0.53±0.75; LS: 0.40±0.90

*Civil et al. [40]*	n=20, 18.1±1.1; females; ballet	39.5±10.8	1.145±0.930

*Nose-Ogura et al. [28]*	n=390, 20.9±4.0; females; mixed (swimming, athletics, gymnastics)	14 % LEA (42/300)	Z-score <−1: 22.7 % (68/300)

*Doyle-Lucas et al. [39]*	n=14, 24.0±1.2; females; control group	40.9	TB: 1.18; LS 0.05
	n=9, 24.3±1.3; females; ballet	5.8	TB: 1.17; posterior-anterior spine Z: 0.56
	n=6, 24.3±1.3; females; ballet	0.6	TB: 0.73; posterior-anterior spine Z: −0.25

*Moore et al. [32]*	n=9, 26.4±4.2; males; running, triathlon, OCR	EA<30	Z-score>−0.9
	n=5, 26.4±4.2; males; running, triathlon, OCR	EA>30	

*Besor et al. [42]*	n=12, 14.3±1.2;females; acrobatic gymnastics – Top position	27±14.3	−0.24±0.93
	n=6, 14.3±2.5; males; acrobatic gymnastics – Base position	39.6±18.5	0.80±0.90 (p=0.032)

**Table 3:** Predicted LEA and BMD.

*Study*	Population (n, age, gender, sport)	Questionnaire used and identified LEA	Z-score
*Vardardottir et al. [23]*	n=26, median 20.2 (17.7–24.5); females;	LEAF-Q≥8	0.93±1.03
	n=30, median 22.4 (17.9–32.8); females;	LEAF-Q<8	1.53±0.89

*Gimunová et al. [38]*	n=12, 23.71±2.94; females; recreational sport	LEAF-Q≥8	TB Z: 0.55 (−0.03–1.20)
	n=12, 23.71±2.94; females; recreational sport	LEAF-Q<8	TB Z: 0.05 (−0.28–0.42)

*Kyte et al. [37]*	n=15, 27.0 (25.0–30.0); females; running (elite athletes)	47 % LEAF-Q≥8	1.7 (1.2–2.3), TB; 0.1 (−0.7–0.6), LS 0.10 (−0.70–0.60), L-spine
	n=15, 26.0 (24.0–28.0); females; control group	13 % LEAF-Q≥8	0.9 (0.8–1.0), TB; −0.1 (–0.5–0.5), LS

*Rogers et al. [24]*	n=112, 19 (15–32); females; various sports	55 % LEAF-Q≥8	LS Z<−1.0: 3 %; Z<−2.0: 1 %; TB Z<−1.0: 2 %

*Civil et al. [40]*	n=20, 18.1±1.1; females; ballet	65 % LEAF-Q≥8	1.145±0.930

*Łuszczki et al. [47]*	n=22, 15.41±1.42; females; football	LEAF-Q ≥ 8	1.15 ± 0.85
	n=12, 15.41±1.42; females; football	LEAF-Q < 8	1.39 ± 0.75

*Rogers et al. [25]*	n=41, 23 (18–32); females; variety of sports	LEAF-Q ≥ 8	LS Z: 0.5±1.2; FN Z: 0.8±1.2

*Rogers et al. [25]*	n=34, 23 (18–32); females, variety of sports	LEAF-Q<8	LS Z: 1.0±1.1; FN Z: 1.3±1.1

*Moore et al. [32]*	n=9, 26.4 ±4.2; males; running, triathlon, OCR	EDI-3: 35.7 % (n=5)	−0.4 up to +0.9 (normal)

*Moore et al. [32]*	n=5, 26.4±4.2; males; running, triathlon, OCR	EDI-3: no risk	−0.4 up to +0.9 (normal)

*Keay et al. [36]*	n=14, age no specified, males; cycling	SEAQ-I (LEA)	−2.0±0.6

*Keay et al. [36]*	n=36, age nonspecified; males; cycling	SEAQ-I negative	−0.4±1.1

*Skorseth et al. [35]*	n=38, 16.9±1.0; females; endurance running	EDE-Q 76.3 % ED	−0.6±1.0 (95 % CI: −0.9 up to −0.3)

*Barrack et al. [27]*	n=30, 15.9±1.1; females; various sports	underweight or amenorrhea	LS: −1.30±1.38 (p<0.001); TB: −0.30±0.98 (p<0.001)

*Barrack et al. [27]*	n=434, 15.7±1.2; females; various sports	no signs of LEA	LS: −0.07±1.21 (p<0.001); TB: 0.53±0.97 (p<0.001)
